# Reinstatement of Eschatoporiini Blaisdell, 1906, a unique tribe of blind cavernicolous Tenebrionidae from California, with a new species from Napa County (Coleoptera, Tenebrionidae, Lagriinae)

**DOI:** 10.3897/zookeys.688.13575

**Published:** 2017-08-10

**Authors:** Rolf L. Aalbu, Kojun Kanda, Aaron D. Smith

**Affiliations:** 1 Department of Entomology, California Academy of Sciences, 55 Music Concourse Drive, San Francisco, CA 94118, USA; 2 Northern Arizona University Department of Biological Sciences 617 S. Beaver St., Flagstaff, AZ 86011-5640, USA

**Keywords:** *Eschatoporis*, Blind, Subterranean, Cave, endemicity

## Abstract

The tribe Eschatoporini Blaisdell, 1906 is reinstated, based on molecular and morphological data, and the spelling corrected as Eschatoporiini. The tribe currently includes only the cave-dwelling genus Eschatoporis Blaisdell, 1906 from California, which is associated with underground aquifers. A second species of Eschatoporis is described from a cave in Napa County, California. The phylogenetic placement of Eschatoporiini within the Lagriinae is examined, and notes on the biology of Eschatoporis are provided.

## Introduction

### Historical background

Blaisdell (1906) described *Eschatoporis nunenmacheri*, a new genus and species of blind tenebrionid collected from under a rock next to a spring. He compared this species to *Eulabis* Eschscholtz, 1829 and *Cerenopus* LeConte, 1851, at that time placed in the Scaurini. Blaisdell suggested that the tribe be expanded to include *Eschatoporis* or that it be placed in a new tribe which he named Eschatoporini. Lacordaire (1859) placed *Eulabis*, *Centrioptera* Mannerheim, 1843, and *Cryptoglossa* Solier, 1836 in his tribe Scaurides Billberg, 1820. *Eulabis* was placed in “Groupe III: Nyctoporides” (Lacordaire 1859: 131) while *Centrioptera* and *Cryptoglossa* were placed in “Groupe IV: Centrioptérides” along with *Cerenopus* (Lacordaire 1859: 135). This is perhaps why Gebien in both his catalogs (1910, 1937) placed *Eschatoporis* in the Cryptoglossini, which now includes the genera *Cryptoglossa* (= *Centrioptera*) and *Asbolus* LeConte, 1851 (= *Cryptoglossa*) see Aalbu (1985, 2005). Despite LeConte’s removal of *Cerenopus* and *Eulabis* from the Cryptoglossini (LeConte 1862), subsequent catalogs listed *Eschatoporis* in the Cryptoglossini.

Aalbu (1985: 50) moved *Eschatoporis* from the Cryptoglossini (subfamily Pimeliinae) to the subfamily Lagriinae, based on morphological data (see discussion below), but placed it as *incertae sedis* within the lagriine tribal classification due to the lack of specimens to dissect at that time. Doyen (1994: 445–446) later tentatively placed *Eschatoporis* in the tribe Goniaderini (Lagriinae). This placement was accepted by Aalbu et al. (2002). Later, Aalbu (2005) placed *Eschatoporis* in the Laenini based on the lack of defensive gland reservoirs and the presence of multiple non-glandular spermathecal tubules. The placement of *Eschatoporis* in the Goniaderini by Aalbu and Smith (2014), as pointed out by Kanda (2016), was an accidental error. At that time, *Eschatoporis* should have remained in the Laenini as pointed out Matthews et al. (2010: 577). These errors, as well as the shuffling of *Eschatoporis* between various tribes, were recently summarized (Kanda 2016), and helped emphasize that a reevaluation of the placement of this genus was overdue.

Over the past decade material belonging to a new species of *Eschatoporis* has been collected from a cave in Napa County, California; thus allowing for a representative of the genus to be sequenced and analyzed within the context of a large pre-existing molecular dataset for the Lagriinae (Kanda et al. 2015). This new species, *Eschatoporis styx*, is described below.

## Materials and methods

For this study, material was borrowed from the following individuals and institutions. These persons (in parentheses) are gratefully acknowledged for loan of their materials:

**ADSC** Aaron Smith Collection, Flagstaff, Arizona, USA (Aaron D. Smith)

**CASC** California Academy of Sciences, San Francisco, California, USA (Dave Kavanaugh).

**CDFA** California State Collection of Arthropods, Sacramento, CA, USA. (Andrew R. Cline)

**NSDA** Nevada State Department of Agriculture, Reno, Nevada, U.S.A. (Robert Bechtel)

**OSAC** Oregon State Arthropod Collection, Corvallis, Oregon, USA. (David R. Maddison)

**RLAC** Rolf L. Aalbu Collection, El Dorado Hills, California, USA. (Rolf L. Aalbu)

## Morphological methods

Measurements were taken using digital calipers or an optical micrometer attached to a Leica MZ16 APO stereomicroscope. Images were taken using a Passport Imaging system (R. Larimer, www.visionarydigital.com). Montaged images were assembled using Zerene Stacker (zerenesystems.com/stacker/) and backgrounds were cleaned up in Adobe Photoshop CS6. Internal structures were cleared with warm 10% KOH and stained with either Chlorazol Black E or Mercurochrome stains.

### Molecular methods

DNA was extracted from a specimen of *Eschatoporis styx* sp. n. collected from the type locality (Clay Cave), using a Qiagen DNeasy Blood and Tissue kit. Four gene fragments were amplified: 28S nuclear ribosomal DNA (28S), arginine kinase (ArgK), carbamoyl phosphate synthetase domain of the rudimentary gene (CAD), and *wingless* (*wg*). These gene fragments were previously sequenced for the Lagriinae sampled in Kanda et al. (2015). Polymerase chain reactions (PCRs) were performed on either an Eppendorf Mastercycler ProS or Mastercycler gradient Thermal Cycler using Ex Taq DNA polymerase (TaKaRa) and basic protocols recommended by the manufacturers. Primer pairs and cycler profiles are described in Kanda et al. (2015). PCR products were cleaned, quantified, and sequenced at the University of Arizona’s Genomic and Technology Core Facility using a 3730 XL Applied Biosystems automatic sequencer. Assembly of multiple chromatograms of each gene fragment and initial base calls were made with Phred v. 0.020425.c (Green and Ewing 2002) and Phrap v. 0.990319 (Green 1999) as orchestrated by Mesquite’s Chromaseq v. 1.12 package (Maddison and Maddison 2014a, 2014b) with subsequent modifications by Chromaseq and manual inspection. Final sequences are available on GenBank (accessions MF370333‒MF370336).

Sequences were incorporated into matrices from Kanda et al. (2015). The final matrix (http://insectbiodiversitylab.org/data/) includes 31 Lagriinae spanning all currently recognized tribes and five outgroup taxa from other subfamilies of Tenebrionidae.

Ribosomal 28S gene fragments were aligned using MAFFT v. 7.130b (Katoh and Standley 2013) and the L-INS-i algorithm. ArgK sequences were manually aligned, asthere were no indels among our sampled taxa. CAD and *wg* were first translated to amino acid sequences, which were aligned using MAFFT v. 7.130b (Katoh and Standley 2013) and the L-INS-i algorithm with default parameter values. The nucleotide sequences were then mapped onto the amino acid alignment using Mesquite (Maddison and Maddison 2014b). The 28S, CAD, and *wg* alignments contained regions with numerous indels. These poorly aligned regions were identified using the server version of Gblocks (Castresana 2000; Talavera and Castresana 2007) with all options for less stringent block selection chosen. For CAD and *wg*, the “Codon” option was selected to maintain the triplet codons in the alignment.

Phylogenetic analyses were performed on a concatenated dataset of all four genes using maximum likelihood (ML), Bayesian (MB), and parsimony (MP) methods. For ML and MB analyses, optimal dataset partitions and substitution models were identified using the BIC implemented in PartitionFinder v.1.1.1 (Lanfear et al. 2012) from initial schemes based on genes and codon position. Two analyses were conducted, first restricting examined models to only those available in RAxML (ML) and then restricting models to only those available in MrBayes (MB). The inferred optimal data partition for ML analyses grouped first and second codon positions of all genes in the first partition, codon position three of ArgK and *wg* in the second partition, codon position three of CAD in the third partition, and 28S in the fourth partition. GTR+I+G was identified as the optimal substitution model for all partitions. The optimal partitioning scheme for MB analyses was the same, but SYM+I+G was identified as the optimal substitution model for the fourth partition.

Maximum Likelihood (ML) analyses were performed using RAxML v. 8.2.9 (Stamatakis 2014) implemented through the Zephyr v. 1.1 package (Maddison and Maddison 2015) in Mesquite (Maddison and Maddison 2014b). Five hundred independent searches for the maximum likelihood tree and 1000 bootstrap replicates were run on all datasets. Bayesian analyses were conducted using MrBayes v. 3.2.2 (Ronquist et al. 2012) on servers maintained by the CIPRES Scientific Gateway (Miller et al. 2010). Analyses were run for 36.8 million generations using default search parameters (two independent runs each with one cold chain and three hot chains). The two runs were considered to have converged when the standard deviation of split frequencies fell below 0.01 and the estimated sample size (ESS) for all parameters was greater than 200, suggesting adequate mixing between the two independent runs. ESS was calculated using Tracer v. 1.6 (Rambaut et al. 2014).

## Taxonomy

Recently, one of us (Kanda 2016) observed what was thought to be tergal defensive gland reservoirs between tergal segments 7 and 8 in *Eschatoporis*, which would be the first example of this reservoir placement in tenebrionids. Whether these cuticular sacs (Kanda 2016, fig. 10) are defensive or not remains unclear as evidence of any defensive secretion was not observed while collecting live specimens. It is possible these may serve another function not as yet determined. Regardless of their function, these cuticular sacs seem to be unique in Tenebrionidae.

Tschinkel and Doyen (1980) examined defensive gland reservoirs, ovipositors, and female genital tubes within Tenebrionidae. In examining female genital tubes, they considered the Adeline lineage the most “primitive” (Tschinkel and Doyen 1980: 337). They found that this condition, where the primary bursa copulatrix gives rises to multiple apical spermathecae and a spermathecal accessory gland was present in all species of both the Adeliini and Pycnocerini (both tribes within Lagriinae) specimens examined. Later, Matthews (1998), in his revision of the genera of Adeliini, found that in some adeliine genera, such as *Isopteron* Hope, 1840, the spermatheca and spermathecal accessory gland are subapical (Matthews 1998: 786). The female reproductive tract of *Eschatoporis* (Fig. 4) can easily fit within the range of the Adeliini in the configuration of both the female internal tract and that the external genitalia lack any of what they termed “advanced” characters. Whether the subapical spermathecae and spermathecal accessory gland represent a small secondary bursa copulatrix (see Matthews 1998: 699) is debatable, but both these characters are found to occur within the Adeliini. However *Eschatoporis* differs from both the Pycnocerini and the Adeliini in lacking sternal defensive glands (Pycnocerini: between segments 7 and 8 or Adeliini: between segments 8 and 9).

This lack of sternal defensive glands, the lack of eyes in some species, as well as the plesiomorphic state of the external female genitalia tract, might place *Eschatoporis* in the Laenini, as some Laenini lack defensive glands. Doyen and Tschinkel (1982: 159) mention that “in Lupropini and Laenini, glands are similar to those of Lagriini and open between sternites 7 and 8” so glands may be present in some Laenini. In any case, the female internal tract of *Laena* (Laenini) differs from *Eschatoporis* in that the spermathecae are few and subapical and the spermathecal accessory gland is apical.

**Figure 1.**
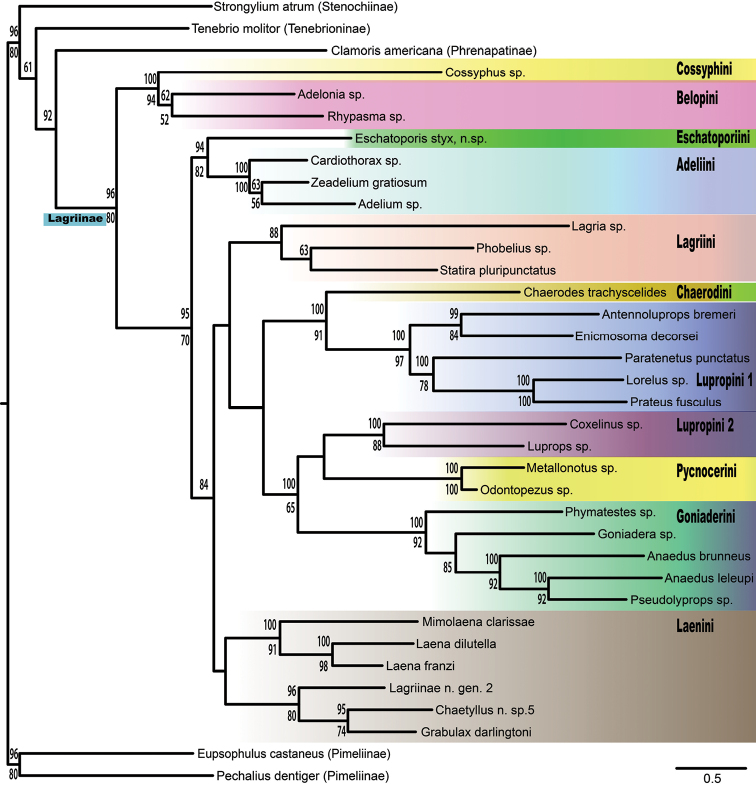
Maximum Likelihood tree from RaxML. Posterior probability values above branches and bootstrap values below. Clades colored according to tribe.

Maximum Likelihood analyses of the 4-gene concatenated dataset recovered *Eschatoporis* sister to a monophyletic Adeliini (Fig. 1). Bootstrap analysis showed moderately high support for this clade (BP = 82). There is no support for the inclusion of *Eschatoporis* in a clade with Laenini. Bayesian analyses converged after 5.85 million generations. The majority rule consensus of post-burn-in trees largely agrees with the ML results. *Eschatoporis* was again recovered as sister to Adeliini (PP=0.94), with no support for its inclusion in Laenini.

### Redefinition of Eschatoporiini

#### Eschatoporiini

Taxon classificationAnimaliaColeopteraTenebrionidae

Blaisdell, 1906

Eschatoporiini Blaisdell, 1906 (Tenebrionidae, Lagriinae)
Eschatoporini Blaisdell, 1906: 78 [stem: Eschatopori-]. Type genus: Eschatoporis Blaisdell, 1906 (type species: Eschatoporis nunenmacheri Blaisdell, 1906, by monotypy). Comment: incorrect original stem formation, not in prevailing usage (See Bouchard et al. 2011: 398).

##### Remarks.

The Eschatoporiini are very similar to the Adeliini and mostly fit within the description and range of the characters as described by Matthews (1998: 701). However, the Eschatoporiini differ from the Adeliini in some key characteristics. The following characters/character states will separate the Eschatoporiini from the Adeliini: Head with basal membrane of labrum exposed, eyeless, but occasionally with a remnant eye scar; maxillary palps with apical segment oblique, not strongly triangular; tentorial bridge present, not arched, sides of tentorium broad, subparallel, continuing to submentum as low ridges; mesepisternum and mesosternum greatly expanded anteriorly forming a neck-like process between thorax and abdomen; scutellum very large; sternal defensive glands absent on all sternites and tergal cuticular sacs present between tergites 7 and 8.

#### Eschatoporis styx

Taxon classificationAnimaliaColeopteraTenebrionidae

Aalbu, Kanda & Smith sp. n.

http://zoobank.org/CEBC2164-969A-4ADC-BB00-BAE5CF85A7FA

Figs 2 , 3 , 4

##### Description.

**Figure 2.**
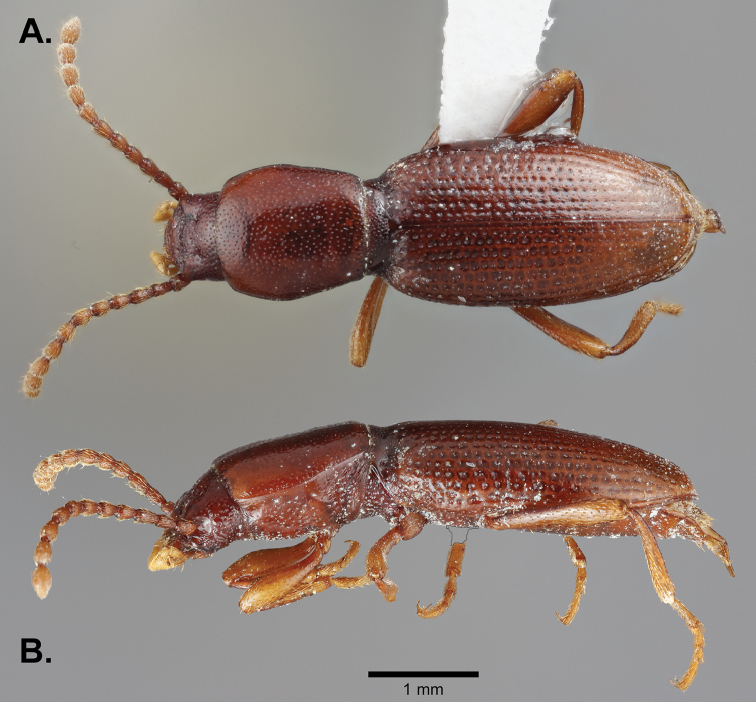
Habitus of *Eschatoporis styx* sp. n.: **A** Dorsal **B** Lateral.

*Holotype male*: Length 5.5 mm. width 1.5 mm. greatest width at mid-elytra. Integument reddish brown, luster slightly shining (Fig. 2).

*Body* elongate, semi-cylindrical, apterous.

*Head* prognathous, widest near base, vertex flattened; surface bearing 1–2 long setae dorso-laterally and few short setae laterally, longer setae moderately long, yellow, approximately twice length of clypeus anteriorly; surface punctuate; distance between punctures about equal to puncture diameter or more, moderate in size, moderately shallow in form; clypeus anteriorly rounded, posteriorly somewhat sinuate, broad, about 4 × as wide as long, bearing two long yellow setae on mid-lateral surface; labrum produced, rectangular, about 1.5 × as broad as long, flattened, with membrane exposed between clypeus and labrum; frons with gena only very slightly produced anteriorly above antennal insertions; eyes absent; mentum square-trapezoid in shape, slightly wider anteriorly; ligula kite shaped, maxillary palps elongate, nearly as long as first four antennomeres, with apical palpomere triangular but hollow apically, interior of apex bearing numerous short setae, ratio of segment lengths 20:10:20:12:21; antennae long and slender, filiform-moniliform, apical segments reaching elytra, eleventh segment longest; ratio of segment lengths 20:16:15:16:15:15:16:17:16:15:26.

*Pronotum* narrower than elytra, subquadrate, slightly arcuate laterally, slightly inflated, widest anterior to middle: anterior margin slightly rounded, posterior surface punctuate, punctures small in size, separated by 1 to 3× puncture diameter, surface glabrous.

*Scutellum* very large, visible, triangular.

*Elytra* only slightly convex, surface punctate-striate, punctures set in 10 even striae on disc, punctures, shallow, moderate in size on disk; distance between punctures approximately equal to puncture diameter; apically, punctures smaller; surface glabrous except few long yellow hairs on apical declivity; three near apex and 1-2 subapically, setae often worn off in older specimens; epipleurae indistinct at base, forming basal part of elytra, only becoming distinct behind metacoxae where elytra abruptly narrows, then gradually narrowing but reaching apex.

*Ventral surface*: prosternal process narrow, convex between procoxae, flattened and slightly expanded posteriorly; mesepisternum and mesosternum greatly expanded anteriorly forming a neck-like process between thorax and abdomen; mesosternum not excavate, distant from prosternal process; mesotrochantin hidden; metacoxae separated by about equal distance between meso-metacoxae; mesocoxae separated by width of coxae; surface of thoracic pleura punctate, interspaced with few moderate sized yellow setae; intercoxal process of abdomen parallel with rounded apex; surface of first visible sternite punctate apically and centrally, punctures becoming smaller more sparse laterally and apically, with few moderate sized yellow setae; second visible abdominal ventrite sparsely, minutely punctate, rest of visible sterna nearly impunctate, with few, small, sparse, shallow punctures; apical sternite with few medium length yellow setae along apex; sternal ratios (anterior to posterior midline) 40:31:25:14:18. Seventh sternite with groove along lateral margin.

*Legs* moderate in length, slender, profemur slightly inflated; leg ratios (femur: tibia) pro. 45:40; meso. 47:37; meta. 65:49; tibiae, tarsi with ventral surface bearing sparse long spine-like setae, femora sparsely setose. Tarsal length ratios as follows (base to apex): protarsus 12:7:5:5:18; mesotarsus 12:10:9:7:21; metatarsus 30:14:9:22.

**Figure 3.**
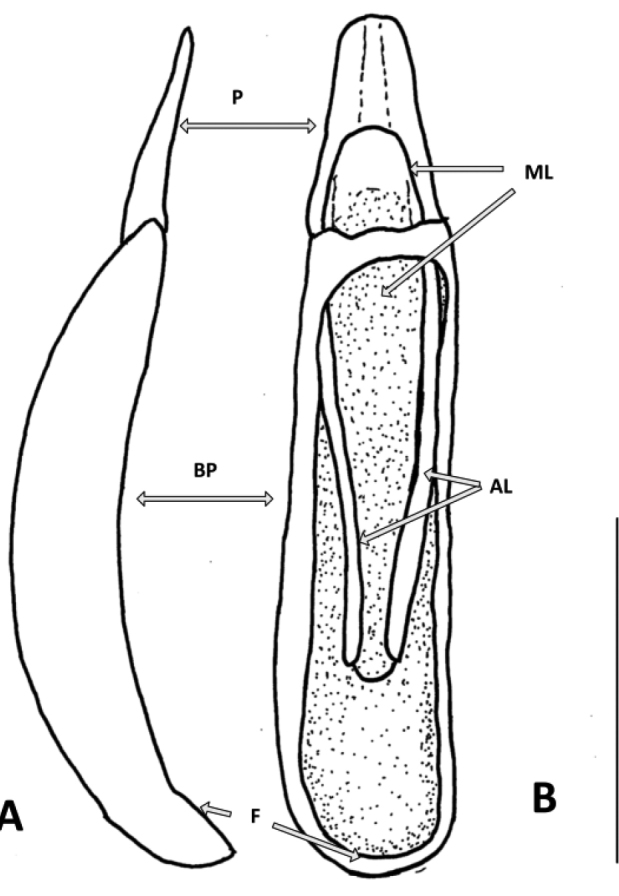
Aedeagus of *Eschatoporis styx* sp. n.: **A** Lateral **B** Ventral. **P** parameres; **AL** ala; **BP** basal piece; **F** flange of basal piece; **ML** median lobe. Scale bar: 0.5 mm.

*Male genitalia*: Aedeagus (Fig. 3) length 1.27 mm., width 0.2 mm. Basal piece elongate, arcuate, with sides not inflected; flange present at base but very small. Parameres short, flat, apex rounded, alae separate, 0.33 mm. Median lobe flat, apex rounded length 0.75 mm, width 0.2 mm.

**Figure 4.**
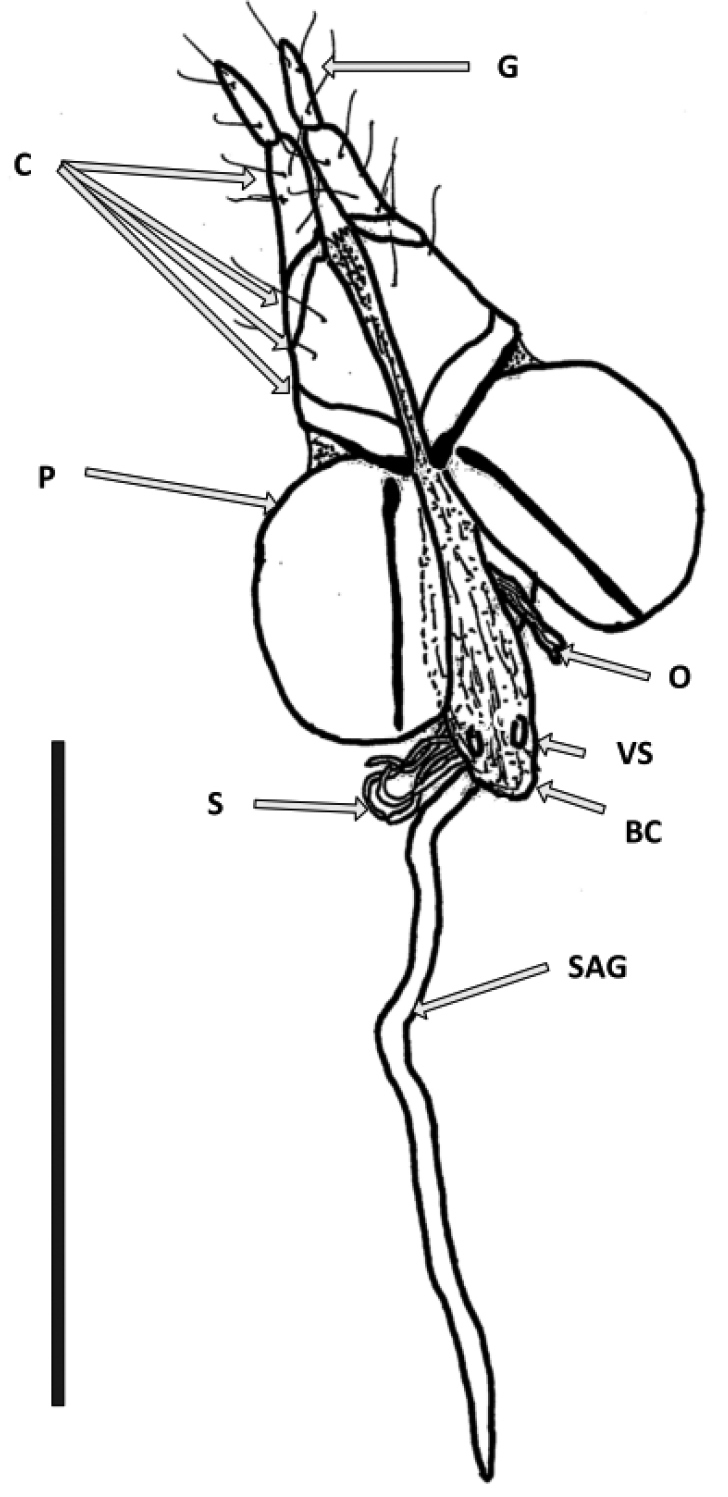
External and internal female genitalia of *Eschatoporis styx* sp. n. Dorsal view. Ovipositor: **G** gonostyle; **C** Coxites; **P** paraproct. Internal genital tract: **O** oviduct; **VS** vaginal sclerites; **S** spermatheca; **BC** bursa copulatrix; **SAG** spermathecal accessory gland. Scale bar: 0.5 mm.

*Allotype female genitalia* (Fig. 4) Ovipositor length 0.5 mm., coxites with segments elongate, slightly longer than paraprocts, gonostyle long and thin. Internal tract with two vaginal sclerites; spermatheca, multiple; spermathecal accessory gland very long and thin, 0.53 mm; spermatheca, spermathecal accessory gland positioned subapically. Bursa copulatrix apical.

*Holotype*: (male) CALIF., Napa Co., White (Clay) Cave, nr. Deer Park, II-26-2005, R. L. Aalbu col. Holoype deposited at CASC.

*Allotype*: (female) CALIF., Napa Co., White (Clay) Cave, nr. Deer Park, II-10-2007, R. L. Aalbu col. Allotype deposited at RLAC.

*Paratypes*: CALIF., Napa Co., White (Clay) Cave, nr. Deer Park, IV-24-2004, R. L. Aalbu col., RLAC (2); same except II-27-2007 (2); same except II-26-2005 (7); same except II-10-2007 (1); same except IV-12-2008 (8); same except VIII-16-2004 (1); same except II-20-2011 (2); same except V-3-2014 (3); same except II-12-2017 (4); same except IV-24-2004, kept alive, found dead VIII-16-2004 (2); same location, collected by K. Kanda and R. L. Aalbu,V-3-2014; Voucher specimen or DNA extraction KKDNA0329.

##### Other material examined

(parts/ condition of specimens not adequate for paratype designation). CALIF., Napa Co. 9 mi. E St. Helena, White Cave, IV-10-1951, Hugh Leech col., CASC (abdomen only) (1); same except White (Clay) Cave, nr. Deer Park, II-26-2005, R. L. Aalbu col., RLAC (1); same except II-10-2007 (5); same except IV-12-2008 (17); same except II-20-2011 (6); same except III-28-2004 (7); same except III-9-2004 (9).

*Larvae*: unknown.

**Figure 5.**
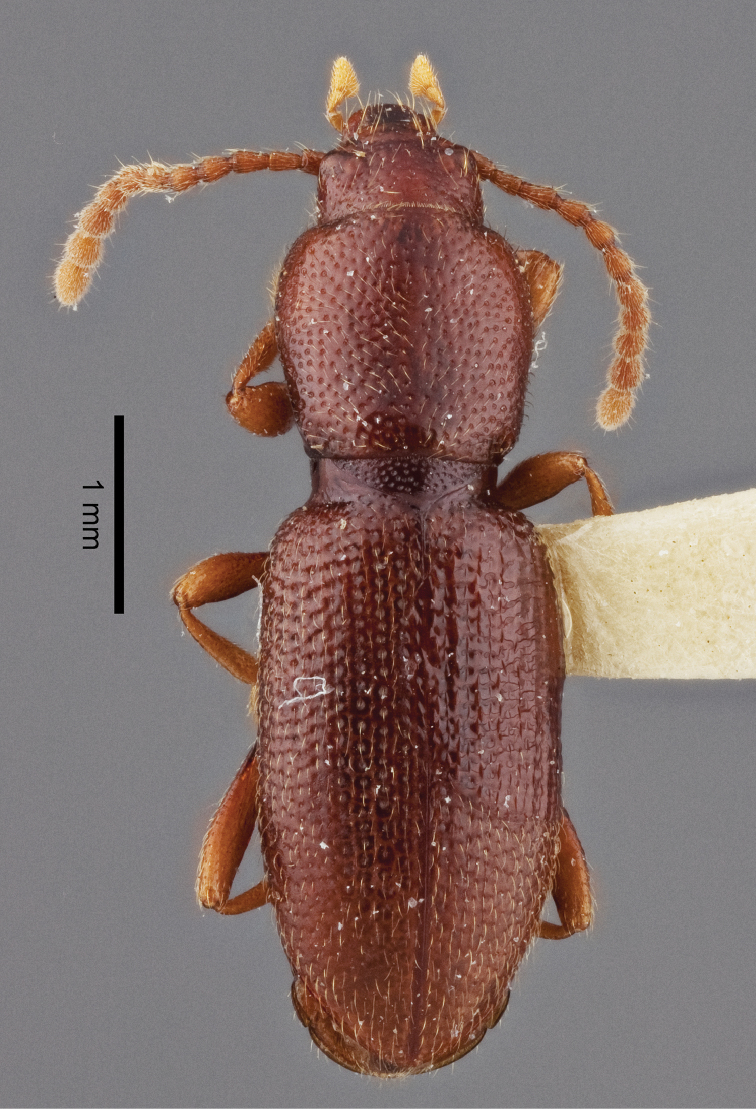
Dorsal habitus of *Eschatoporis nunenmacheri* Blaisdell.

The two species of *Eschatoporis* can easily be separated by the clearly different setation patterns on the elytra. While in *E. nunenmacheri*, the elytra are covered with short setae (Fig. 5), in *E. styx*, (Fig. 2) the elytra are glabrous except for a few long, hair-like setae near and at the base of the elytra. *Eschatoporis styx*, also lacks any eye “scar” which is found in various sizes in *E. nunenmacheri*.

#### Eschatoporis nunenmacheri

Taxon classificationAnimaliaColeopteraTenebrionidae

Blaisdell, 1906

Fig. 5

##### Material examined.

CALIF., Marin Co. Mill Valley, I-18-1948 (CDFA, 1); same except V-3-1947, E. S. Ross, In rock crack 4’ below surface, *Eschatoporis nunenmacheri* det. Aalbu, 2004 ((NSDA, 1); CALIF., Marin Co. Fairfax, IV-6-1919, Van Dyke Colln. (CASC, 1); same except V-25-1919, (CASC, 1); CALIF., Marin Co. Samuel P. Taylor St. Pk. II-3-1958 J. Helfer (CASC, 1); same except XII-13-1954, *Eschatoporis nunenmacheri* det. Boddy, 1955, (1); same except South Entrance, XI-3-1953 G. A. Marsh, R. O. Schuster cols., *Eschatoporis nunenmacheri* det. Boddy, 1955, (2).

It is unclear from Blaisdell’s description (1906) where the Holotype (California, Marin Co., Fairfax, June, collected by Nunenmacher while digging on a ledge near a spring) was deposited. Checks of the CASC, Philadelphia Academy of Sciences, Museum of Comparative Zoology, Harvard University and Smithsonian did not locate the type. However, from Blaisdell’s description and drawing, it is clear that the holotype is the same as the other specimens of *Eschatoporis nunenmacheri*.

### Discussion and notes on biology

Clay Cave is located in oak woodland in the California wine country adjacent to the northern margin of San Francisco Bay, California. Known since the 1870s, the cave formed as a soil pipe cave in an ash flow of the Miocene Sonoma Volcanics, a continental packet of rhyolitic to andesitic volcanoclastic sediments and tephras. The cave consists of 229 m of linear passage with several small rooms floored with a seasonal stream (see Elliott et al., in press: fig.17). It appears that this cave originated along root casts in the bedded volcanic sediments that are mostly altered to smectite clay locally stained with iron oxides. Subsequent invasion by seasonal streams has integrated the initial fist-sized soil pipes into vadose canyon passages. The cave has at least two seeping springs. Clay Cave also has a rich biota, including some unusual terrestrial invertebrates, and is ranked fourth in the most bio-diverse caves of California (Elliott et al. in press).

Repeated attempts to find larvae in the cave or acquire larvae from adults in the lab yielded no results.

Species of the tribe Eschatoporiini seem to be associated with deep interstitial layers in rocky soils or underground water flows. Specimens of the tribe are either collected in deep rocky soil layers or in caves, both near springs. In Clay Cave, most *Eschatoporis styx* were collected under rocks rather than walking freely. Sometimes specimens have been found dead in standing small pools water from spring seepage in the cave. Specimens of Eschatoporiini remain very rare in collections. For instance, as far as we know *Eschatoporis nunenmacheri* has not been recollected since 1958.

*Eschatoporis* species are very similar in appearance and biology to the laenine genus *Hypolaenopsis* (Masumoto, 2001), which was originally placed in Adeliini but subsequently transferred to Laenini (see Schawaller 2008). Some *Hypolaenopsis* species are superficially very similar to *Eschatoporis*, differing only in size. The species *H. nanpingica* (originally described in *Laena* by Schawaller 2001), which is blind with only an eye scar remaining (as in *E. nunenmacheri*), could be mistaken for a large species of *Eschatoporis* based on external morphology. Other *Hypolaenopsis* species also have reduced eyes. Even the species-rich genus *Laena* contains taxa with reduced eyes; such as *L. subcoeca* Kaszab, 1973 and *L. sherpa* Schawaller, 2002 (both from forest litter in Nepal), and *L. deplanata* Weise, 1878 from Turkey in which the eyes are reduced to single facets (Schwaller, personal communication).

Masumoto (2001) mentioned that all specimens of *Hypolaenopsis nanpingica* were taken from the upper hypogean zone by digging soil mingled with gravel beneath large stones to the depth of 20–30 cm, about five or six meters above a stream.

## Supplementary Material

XML Treatment for Eschatoporiini

XML Treatment for Eschatoporis styx

XML Treatment for Eschatoporis nunenmacheri
